# Skeletal muscle mass associates with pancreaticoduodenectomy operative time in a sex-dependent manner

**DOI:** 10.1186/s12885-025-15055-2

**Published:** 2025-12-29

**Authors:** Sophia Xiao, Ashley Freeman, Emily Kalmanek, Kelsey Steckly, Mary Belding-Schmitt, Carlos H.F. Chan, Erin E. Talbert

**Affiliations:** 1 Carver College of Medicine, University of Iowa, Iowa City, IA 52242 USA; 2 Department of Health, Sport, and Human Physiology, University of Iowa, 375 Newton Road, Iowa City, IA 52242 USA; 3 Holden Comprehensive Cancer Center, University of Iowa, Iowa City, IA 52242 USA; 4 Department of Surgery, University of Iowa, Iowa City, IA 52242 USA

**Keywords:** Whipple procedure, Operative time, Body composition, Pancreatectomy, Skeletal muscle, OR efficiency, OR utilization

## Abstract

**Background:**

Pancreaticoduodenectomy, also known as the Whipple procedure, is a complex surgery for which increased operative time is associated with worse outcomes for patients. Body composition has been shown to be a contributing factor to operative time and can vary widely amongst pancreaticoduodenectomy patients. We hypothesized that greater amounts of adipose tissue are associated with extended pancreaticoduodenectomy operative time.

**Methods:**

Demographic variables were retrieved retrospectively from the medical record for the first 211 consecutive patients enrolled in an institutional biobanking protocol with malignancies associated with pancreatectomy. Our final cohort of 68 patients underwent a pancreaticoduodenectomy and had preoperative CTs available for body composition analysis. Variables of interest were associated with operating time.

**Results:**

Younger patient age, greater number of lymph nodes removed, and the need for a vascular repair were all associated with increased operative time. When considering surgeries without vascular repairs (*n* = 56), neither subcutaneous adipose (*p* = 0.80) nor visceral adipose (*p* = 0.32) were associated with surgery length. Skeletal muscle was unique, with greater muscle mass tending to associate with longer operating times (*p* = 0.051). Additionally, a sexual dimorphism was revealed whereby increased operative time was associated with greater skeletal muscle mass for females (*p* = 0.005) but lower skeletal muscle mass for males (*p* < 0.001).

**Conclusions:**

Contrary to expectations, increased adiposity was not associated with extended pancreaticoduodenectomy operative time. However, skeletal muscle mass was associated with operative time in a sex-dependent matter. Assessment of skeletal muscle mass could prove useful in identifying patients at risk of prolonged pancreaticoduodenectomy operations.

Pancreaticoduodenectomies, commonly known as Whipple procedures, are major surgeries performed for various gastrointestinal diseases, with the most common indication being resectable malignancy of the pancreas [1]. Obesity, defined as a body mass index (BMI) of greater than 30 kg/m^2^, predisposes patients to the development of pancreatic malignancy [2, 3]. Therefore, many patients in need of a pancreaticoduodenectomy carry excess adipose tissue. However, pancreatic malignancy often results in severe weight loss, including the loss of skeletal muscle, called cachexia [4]. Therefore, pancreaticoduodenectomy patients are at high risk of skeletal muscle mass loss and sarcopenic obesity [5–7], and the body compositions of patients requiring pancreaticoduodenectomy have nuances specific to their disease.

It is well-known that in patients undergoing pancreatectomy, higher BMI is associated with greater intraoperative blood loss and postoperative complications [8–10]. Longer operative times for pancreaticoduodenectomy patients are also associated with worse perioperative outcomes and shortened long-term survival [11–13]. Although body composition can significantly impact surgical time across all procedures, with each BMI unit increasing operative time by 2–3% [14], the data on the impact of BMI on pancreaticoduodenectomy operative time is mixed [8, 9, 15–19].

Given the conflicting data on the effect of body composition on pancreaticoduodenectomy operative time and the limitations of BMI in assessing adiposity [20], our study aimed to harness individual body compartment data to dissect the relationship between body composition and operative time. Although many measurement methods exist, cross-sectional imaging is commonly used to estimate total body lean muscle and adipose tissue mass in people with cancer, as use of existing images allows body composition to be determined without burdening patients with an additional test [21, 22]. This method is particularly useful for patients with abdominal cancers, as they undergo frequent routine scans for tumor assessment.

The existing data relating operative variables and isolated body composition components are limited. In patients with reduced muscle mass, studies have shown that resection rate and long-term post-surgical survival worsen [23–25], but the impact of muscle mass on operative time is unknown. Furthermore, adipose tissue is a component of weight loss in patients undergoing pancreatectomy, with loss of both visceral and subcutaneous adipose beginning prior to diagnosis [26]. However, the impact of adipose mass on operative time has not been characterized but is anecdotally assumed to extend the length of surgeries. In this study, we hypothesized that greater amounts of adipose tissue would be associated with extended operative time.

Given the length of pancreaticoduodenectomies, the poor outcomes associated with longer surgeries, and the high cost of operating room (OR) time [27], there is great interest in understanding the factors that contribute to longer surgeries. Understanding the drivers of prolonged operative time could identify opportunities to shorten operative time and improve patients’ outcomes during and after their perioperative period. For the hospital, more accurate estimates of operative time duration would also allow for greater optimization of OR Block Utilization and OR Prime Time Utilization, which are key metrics for assessing inefficiencies and improving productivity and resource allocation within complex health care systems.

## Methods

### Study cohort

Patients with cancer receiving care at the University of Iowa Health Care are approached to consent to research under the PERCH (Patients Enhancing Research Collaboration at Holden) program at the Holden Comprehensive Cancer Center. Gastrointestinal cancer patients consent to PERCH through the Gastrointestinal Molecular Epidemiology Resource, which has been approved by the University of Iowa Institutional Review Board (IRB # 201202742, first approved February 21, 2012).

The research herein was conducted with the data of patients who provided informed consent to participate PERCH and related research. This specific research was approved under a waiver of consent by the University of Iowa Institutional Review Board (IRB # 202101083, first approved July 9, 2021), with appropriate protections of patient privacy in compliance with the Declaration of Helsinki.

**Fig. 1:**
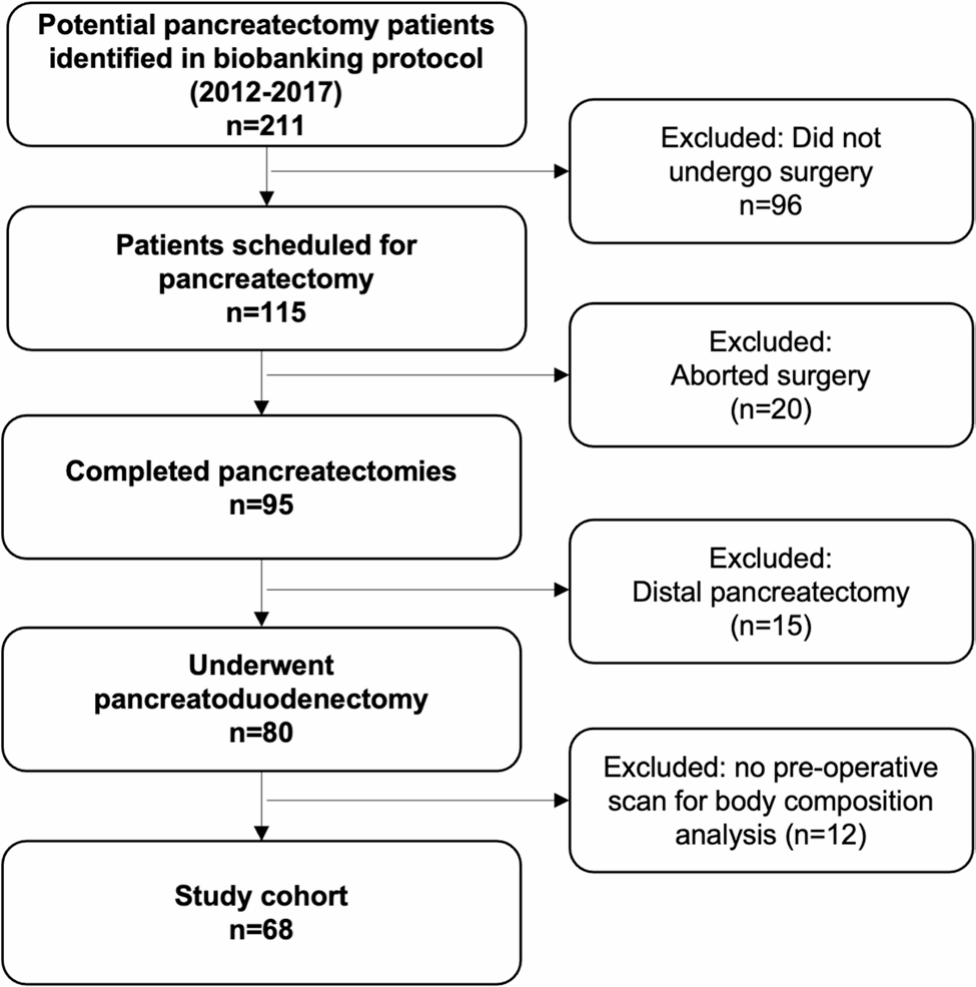
Participant CONSORT table

This retrospective study includes patients enrolled in PERCH between 2012 and 2017. The first 211 consecutive participants with gastrointestinal malignancies associated with pancreatectomy were assessed for inclusion, and cases were included if patients underwent a completed pancreaticoduodenectomy and had a pre-operative CT scan available for analysis (Fig. 1).

### Medical record review

Demographic variables of interest, cancer type, anti-cancer therapy prior to surgery, and BMI at the time of surgery were retrieved from the medical record. Resectability was determined based upon version 2.2025 of the National Comprehensive Cancer Center (NCCN) Pancreatic Adenocarcinoma guidelines [28]. Performance status was assessed via American Society of Anesthesiologists (ASA) physical classifications [29]. Body surface area (BSA) was calculated by the Mosteller method [30]. Operative times were determined from National Surgical Quality Improvement Program (NSQIP) reports or calculated from the procedure encounter. The need for a vascular repair was determined from the operative note. Number of retrieved lymph nodes and T, N, and M stages were retrieved from surgical pathology reports. Tumor staging was standardized to the 8th edition of the AJCC Cancer Staging Manual [31].

### Assessment of body composition

Body composition was quantified using preoperative CT scans at lumbar vertebrae 3. Cross-sectional surface areas (cm^2^) of skeletal muscle, visceral adipose tissue, and subcutaneous adipose tissue were analyzed using SliceOmatic software Version 5.0 (Tomovision, Montreal) and the Automated Body Composition Analyzer using Computed Tomography Image Segmentation (ABCAS, Voronoi Health Analytics Inc., Vancouver) using established Hounsfield unit (HU) radiodensity thresholds (skeletal muscle: −29 to + 150; subcutaneous adipose: −190 to −30; visceral adipose: −150 to −50) [32]. Segmentation of a single slice at L3 was visually assessed by a blinded investigator, and any clear errors were manually corrected. Cross-sectional area of the segmented tissues was summated and then height-normalized to estimate tissue indices, a common and accepted measurement [21, 33]. Specifically, skeletal muscle index was calculated as [skeletal muscle area/height^2^]; subcutaneous adipose tissue index was calculated as [subcutaneous adipose area/height^2^]; and visceral adipose tissue index was calculated as [visceral adipose area/height^2^]. The resulting values are all expressed in cm^2^/m^2^.

### Statistical analyses

Operative time was compared between two groups divided by relevant variables using a Mann-Whitney test for non-normally distributed data or Welch’s t-tests for normally distributed data without equal standard deviations. For three groups, a one-way ANOVA was utilized with Tukey’s multiple comparisons test. Relationships between variables of interest and operative time were assessed using univariable linear regression. Multivariable regression and partial correlations were used to adjust for factors resulting in extended operative times. Alpha was set a priori at *p* = 0.05. All analyses were performed with SPSS Version 29, and figures were created using GraphPad Prism 10.

### Data availability

The datasets used in the current study are not publicly available due to the relatively small sample size and risk of identification. They are available from the corresponding author on reasonable request and approval of the University of Iowa Institutional Review Board, as the original ethics approval did not include approval to publish individual datapoints.

## Results

### Study population characteristics

A total of 68 patients who underwent a pancreaticoduodenectomy (Whipple procedure) between 2012 and 2017 were identified for the study (Table 1). Patients had an average age of 64 years, and slightly more patients were male (52.9%). Most patients had a diagnosis of pancreatic adenocarcinoma (*n* = 65, 94.1%), with 3 patients diagnosed with cholangiocarcinoma. Most patients were assessed to have tumor stage T2 (*n* = 39, 57.4%), with disease-positive lymph nodes (stage N1, *n* = 28, 41.2% or stage N2, *n* = 20, 29.4%), and without metastatic disease (M0/Mx, *n* = 66, 97.1%). Across patients, mean procedure duration was 453.0 ± 108.9 min, with a median procedure duration of 432 min.

**Table 1:** Patient characteristics

**Variable**	**Patients (n=68)**	**Percentage (%)**
Age (years ± SD)	64.5 ± 10.1	
Height (cm ± SD)	171.6 ± 9.9	
Weight (kg ± SD)	84.2 ± 42.4	
Duration of surgery (minutes)		
Median (range)	432 (273 - 759)	
Mean (± SD)	453.0 ± 108.9	
Sex		
Female	32	47.1
Male	36	52.9
Disease Histologic Subtype		
Pancreatic adenocarcinoma	65	94.1
Cholangiocarcinoma	3	5.9
ASA Classification		
1	1	1.5
2	23	33.8
3	41	60.3
4	3	4.4
Resectability		
Resectable	54	79.4
Borderline Resectable	14	20.6
Neoadjuvant Therapy		
Yes	18	26.5
No	50	73.5
Neoadjuvant Radiation		
Yes	7	10.3
No	61	89.7
T stage		
T1	17	25.0
T2	39	57.4
T3	12	17.6
N Stage		
N0	20	29.4
N1	28	41.2
N2	20	29.4
M Stage		
M0/Mx	66	97.1
M1	2	2.9
Lymph Nodes Removed		
Median (range)	18 (1 - 47)	
Mean (± SD)	18.3 ± 8.6	
Vascular Repair		
Yes	12	17.6
No	56	82.4
Surgeon		
A	32	47.0
B	17	25.0
C	18	26.5
D	1	1.5

### Impact of age, sex, and performance status on operative time

**Fig. 2:**
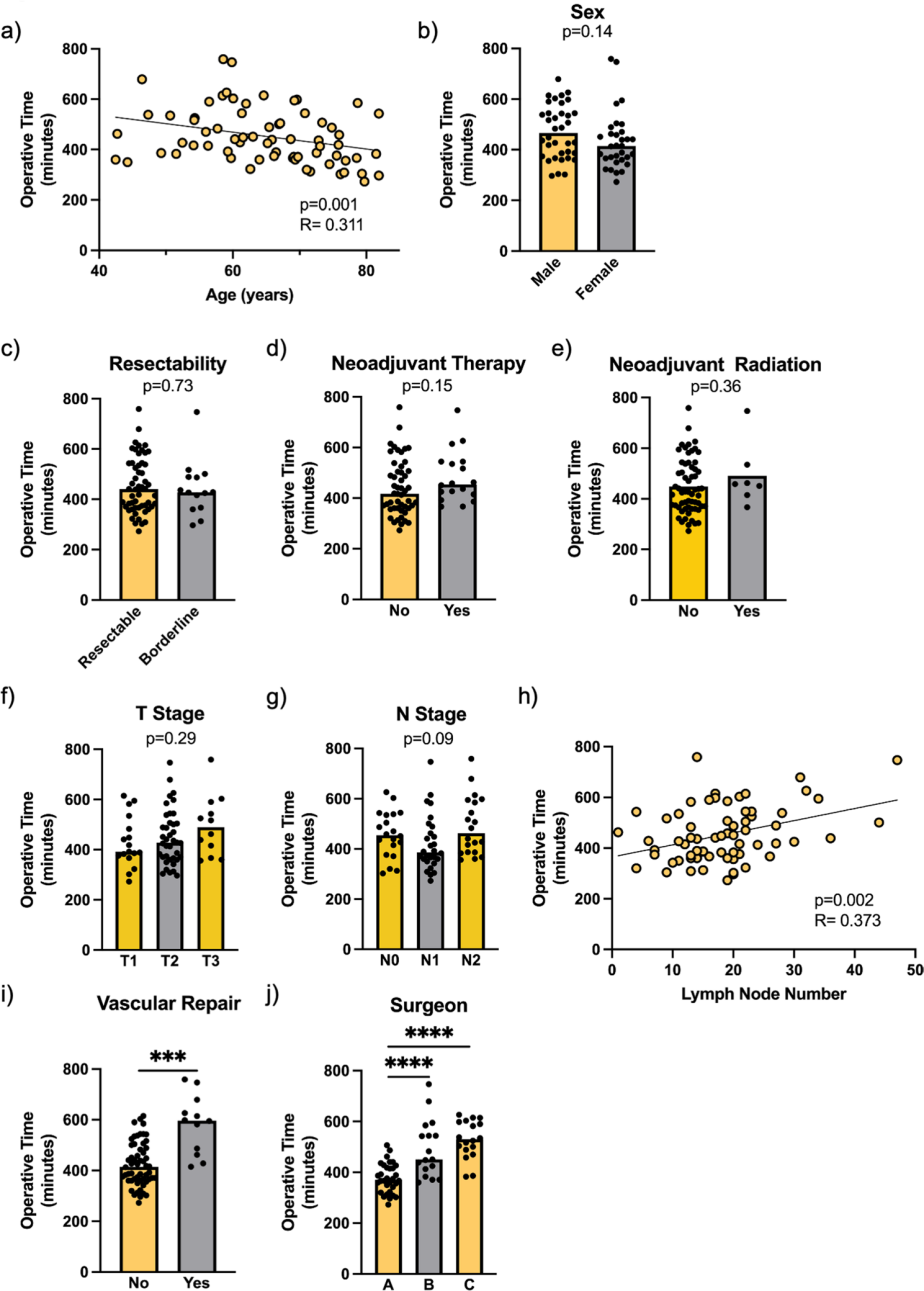
Perioperative Variables and Operative Time. **a** Advanced age was associated with shorter operative time (*p*=0.001) (b) Operative time did not differ between male and female patients (*p*=0.140) (**c**). Operative time did not differ between borderline resectable and resectable patients (*p*=0.734). **d**-**e** Operative time did not significantly differ with neoadjuvant therapy (*p*=0.149) or neoadjuvant radiation (*p*=0.364). **f**-**g** Operative time did not differ with T stage (*p*=0.287) or between N0 vs N1 vs N2 stage patients (*p*=0.086). **h** Longer operative time was significantly associated with greater number of lymph nodes removed (*p*=0.002). **i** Vascular repair (*n*=12) increased operative time (p<0.0005, indicated by ***). **j** Operating surgeon had a significant impact on operative time. *p*<0.0001, indicated by ****. *n*=68 except Fig. 2**j**, *n*=67.Perioperative Variables and Operative Time. **a** Advanced age was associated with shorter operative time (*p*=0.001) (**b**) Operative time did not differ between male and female patients (*p*=0.140) (**c**) Operative time did not differ between borderline resectable and resectable patients (*p*=0.734). (**d**-**e**) Operative time did not significantly differ with neoadjuvant therapy (*p*=0.149) or neoadjuvant radiation (*p*=0.364). (**f**-**g**) Operative time did not differ with T stage (*p*=0.287) or between N0 vs N1 vs N2 stage patients (*p*=0.086). **h** Longer operative time was significantly associated with greater number of lymph nodes removed (*p*=0.002). **i** Vascular repair (*n*=12) increased operative time (*p*<0.0005, indicated by ***). **j** Operating surgeon had a significant impact on operative time. *p*<0.0001, indicated by ****. *n*=68 except Fig. 2**j**, *n*=67

Previous work has established that pancreaticoduodenectomy time is impacted by patient age and performance status. Specifically, operative time is reduced for patients older than 65 years of age and increased for patients with an ASA score of greater than or equal to 3 [34]. We found no association between ASA score and operative time in our cohort (*p* = 0.77), but consistent with previous findings, shorter operative time was associated with increasing age (Fig. 2a, *p* < 0.001). Operative time was not impacted by sex, as there was no difference between male and female patients (Fig. 2b, *p* = 0.14).

### Impact of perioperative treatment and disease stage on operative time

There was no significant difference in operative time between tumors classified as resectable or borderline resectable (*p* = 0.73, Fig. 2c), nor was there any difference in operative time between patients receiving and not receiving any neoadjuvant therapy (*p* = 0.15, Fig. 2d). While previous work has shown neoadjuvant radiation to increase operative time [35], our data demonstrated no significant impact on overall operative time from neoadjuvant radiation therapy (*p* = 0.36, Fig. 2e). T stage did not impact surgery duration (*p* = 0.29, Fig. 2f). The malignancy stage of the removed lymph nodes (N0 vs. N1 vs. N2) trended towards significance, but no post-hoc comparison between groups demonstrated an impact on procedure duration (N1 versus N2, *p* = 0.07, Fig. 2g). However, a greater extent of lymph node dissection was associated with increased operative time (*p* = 0.002, Fig. 2h), consistent with previous studies [24, 36].

Surgeries requiring vascular repairs had increased operative times (*p* = 0.0005), with 12 surgeries involving vascular repairs (Fig. 2i). Finally, we considered the impact of individual surgeons. All but one operation was performed by one of three surgeons, and for these 67 surgeries, operative time was significantly lower for one surgeon compared to the other two surgeons (Fig. 2j, *p* < 0.0001).

### Impact of body composition on operative time

**Fig. 3:**
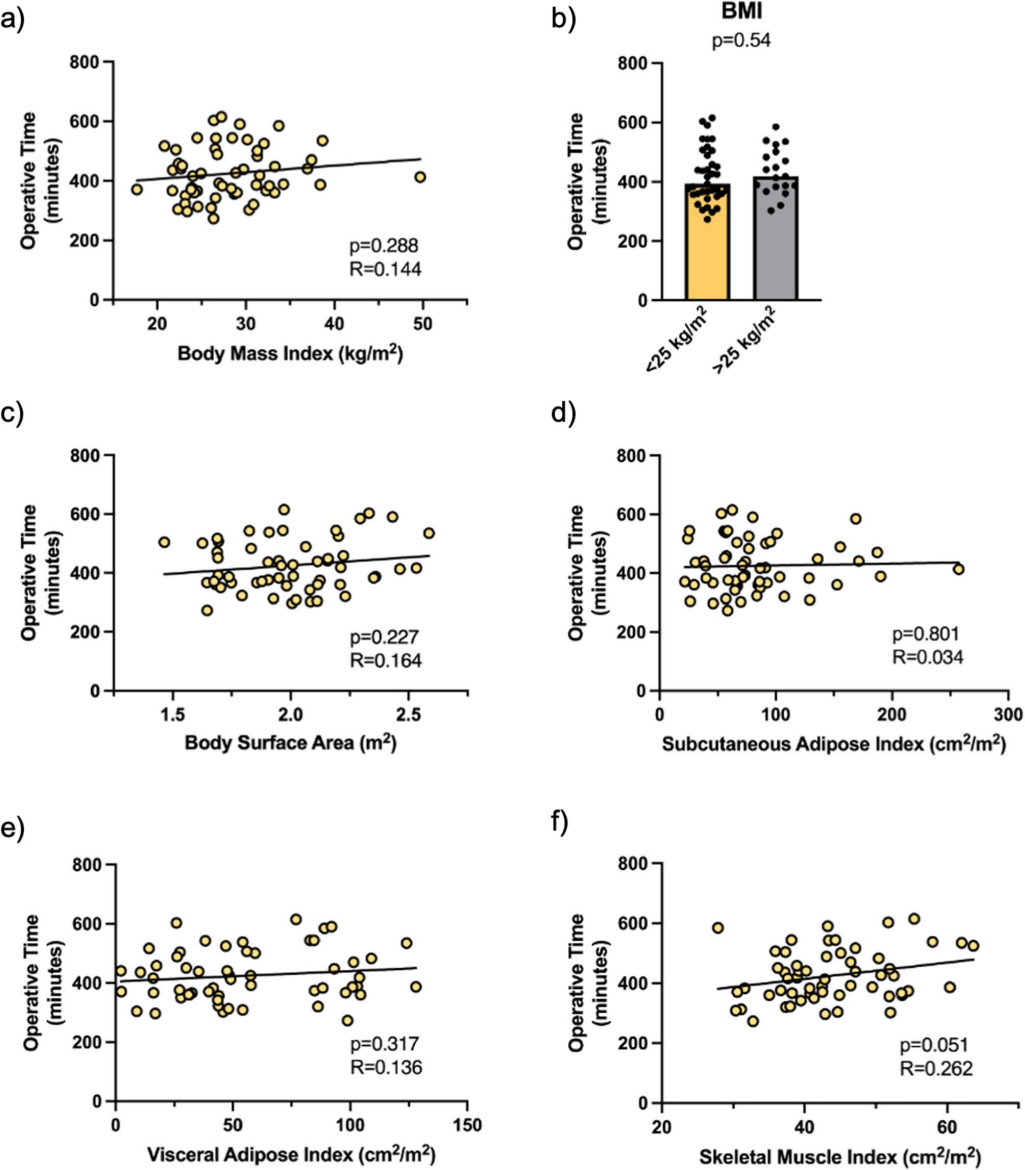
Body composition and operative time in patients not requiring a vascular repair. **a** Association of operative time with body mass index (BMI, *p*=0.228). **b** BMI greater than or equal to 25 kg/m2 was not associated with longer operative time (*p*=0.543). **c** Association between operative time and body surface area (BSA) (*p*=0.227) (**d**-**e**) Association between operative time and subcutaneous adipose index (*p*= and visceral adipose index (*p*=0.317), (**f**) Association between operative time and skeletal muscle index (*p*=0.051) *n*=56. Body composition and operative time in patients not requiring a vascular repair. **a** Association of operative time with body mass index (BMI, *p*=0.228). **b** BMI greater than or equal to 25 kg/m2 was not associated with longer operative time (*p*=0.543). **c** Association between operative time and body surface area (BSA) (*p*=0.227). **d**-**e** Association between operative time and subcutaneous adipose index (*p*= and visceral adipose index (*p*=0.317), **f** Association between operative time and skeletal muscle index (*p*=0.051) *n*=56

Because of the unpredictable nature of vascular repairs, we focused our investigation into the role of body composition on patients not requiring a vascular repair (Table 2). For these 56 patients, we did not find a significant association between BMI and increased operative time (*p* = 0.29, Fig. 3a). Previous work has identified that patients with a BMI equal to or greater than 25 kg/m^2^ have longer pancreaticoduodenectomy operative times [18], but this was not true in our dataset (*p* = 0.54, Fig. 3b). Furthermore, BSA (*p* = 0.23) was not correlated with longer operative times (Fig. 3c).

**Table 2:** Body composition of patients not undergoing vascular repairs

Variable	All Patients (*n* = 56)	Females (*n* = 26)	Males (*n* = 30)
BMI (kg/m^2^ ± SD)	28.17 ± 5.56	27.85 ± 6.66	28.44 ± 4.50
Body Surface Area (m^2^ ± SD)	1.99 ± 0.26	1.97 ± 0.27	2.02 ± 0.24
Subcutaneous Adipose Index (cm^2^/m^2^ ± SD)	83.13 ± 47.34	98.97 ± 56.83	69.40 ± 32.34
Visceral Adipose Index (cm^2^/m^2^ ± SD)	55.85 ± 33.69	48.27 ± 30.75	62.41 ± 35.24
Skeletal Muscle Index (cm^2^/m^2^ ± SD)	43.77 ± 8.33	38.67 ± 4.74	48.19 ± 8.30

To test our hypothesis regarding an association between increased adiposity and operative time, we normalized adipose tissue areas measured on CT images to height^2^, called an adipose tissue index. Contrary to our prediction, neither subcutaneous adipose index (Fig. 3d, *p* = 0.80) nor visceral adipose index (Fig. 3e, *p* = 0.32) were associated with operative time. Surprisingly, greater height-normalized muscle mass as characterized by skeletal muscle index approached significance with longer operative times (Fig. 3f, *p* = 0.051).

Based on our findings in Fig. 2, we adjusted body composition variables for surgeon, lymph node number, age, and sex. Adjustment largely did not change the relationships between body composition and operative time, confirming that subcutaneous adipose tissue, visceral adipose tissue, BSA, and BMI are likely not contributing to increased pancreaticoduodenectomy times (Table 3). Adjustment strengthened the relationship between skeletal muscle mass and operative time (*p* = 0.010).

**Table 3:** Relationships between body composition variables and operative time

	Unadjusted			Adjusted		
	β	*R*	*p* value		*R*	*p* value
Body Mass Index	2.254	0.144	0.288	0.323	0.031	0.826
Body Surface Area	55.489	0.164	0.227	34.568	0.156	0.268
Subcutaneous Adipose Index	0.063	0.034	0.801	0.194	0.154	0.276
Visceral Adipose Index	0.351	0.136	0.317	−0.029	−0.017	0.907
Skeletal Muscle Index	2.726	0.262	0.051	−3.612	−0.352	0.010*

Adjustment: Surgeon, Lymph Nodes Removed, Age, and Sex

### Sex-dependent association between skeletal muscle mass and operative time

**Fig. 4:**
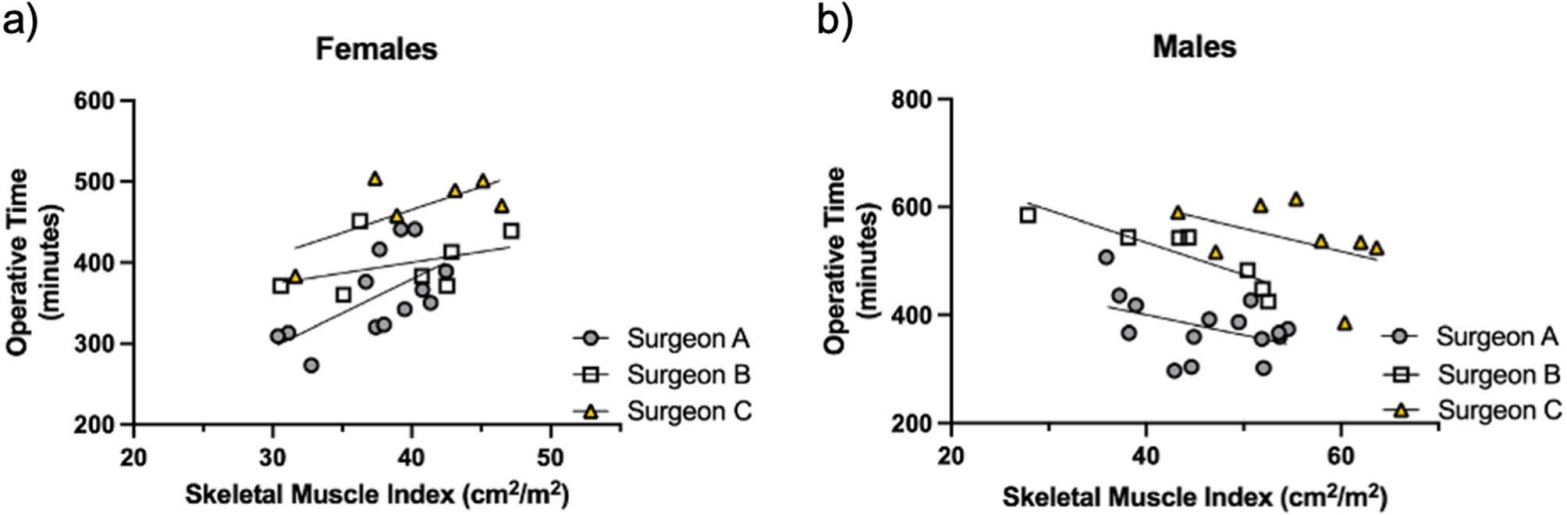
Skeletal Muscle Index is related to operative time in a sexually dimorphic manner. **a** Females *n*=26; **b** Males *n*=30. Statistical relationships appear in Table 4, including adjustments by surgeon. All surgeries were performed by one of three surgeons

Although adjustment for age, surgeon, lymph node removal, and sex strengthened the relationship between operative time and skeletal muscle mass, it also inverted the relationship. Furthermore, the inclusion of sex was required for an association. Therefore, we hypothesized that skeletal muscle mass may be associated with altered operative time in a sex-dependent way and dichotomized our data by sex. Given the relatively small sample size, we only adjusted for surgeon, which had by far the largest impact on operative time of our associated variables. For females, increased operative time was associated with greater skeletal muscle mass (*p* = 0.005, Table 4; Fig. 4a). In contrast, lower skeletal muscle mass was associated with increased operative time for males (*p* < 0.001, Table 4; Fig. 4b).

**Table 4:** Relationship between operative time and body composition variables is sexually dimorphic

	Unadjusted			Adjusted		
	β	*R*	*p* value	β	*R*	*p* value
Females	7.495	0.562	0.003	5.453	0.546	0.005
Males	−0.672	−0.058	0.761	−5.047	−0.607	< 0.001

Adjustment: Surgeon

## Discussion

Pancreaticoduodenectomies are long, technically demanding surgeries that often require challenging intraoperative decisions. The complexity of the procedure is increased by the patient’s health status, disease process, surgeon experience, and institutional factors. Furthermore, most pancreaticoduodenectomies are conducted for malignancies, and the extent of cancer, pre-operative history, and intraoperative factors may also impact a surgeon’s decision on the extent of lymphadenectomy, which impacts operative time. Because of the length and unpredictable nature of these surgeries, there is significant interest in more effectively predicting operative times to maximize operating room utilization. Additionally, longer operations are associated with increased morbidity, complications, and length of hospitalization after pancreatic resection; perioperative outcomes potentially could be improved by additional efforts to reduce operative times that are anticipated to be longer [34, 37, 38].

The mean and median operative times found in our study were longer than those published by other institutions, which were closer to 5.5 h compared to our 7.2 h [1, 11, 39–41]. Our longer operative times may be in part because our study population was derived from a cancer biobank and malignancy was the cause of surgery, while most other studies cited a number of causes for pancreaticoduodenectomies.

To our knowledge, no other study has assessed the relationship between pancreaticoduodenectomy operative time and adiposity. Contrary to our hypothesis, neither subcutaneous nor visceral adipose tissue mass were associated with operative time. Furthermore, neither BMI nor BSA were associated with increased operative time. Our findings are consistent with the existing literature in which pancreaticoduodenectomy surgical duration has not been found to be associated with BMI [16, 17]. Some studies have identified BMI cut-off points above which patients with greater BMI (>25 kg/m^2^ or >35 kg/m^2^, depending on study) had extended operative compared to other patients [9, 15, 18], but this was not true in our study.

Surprisingly, we identified a sexual dimorphism in the relationship between operative time and muscle mass. When considered separately, each additional unit of skeletal muscle mass (cm^2^/m^2^) was associated with either five and a half more minutes of operative time for female patients or five fewer minutes of operative time for males. For males, skeletal muscle index ranged from 27.9 to 63.7 cm^2^/m^2^, which equates to a range of 3 h of operative time. For females, with a range of 30.4 to 47.2 cm^2^/m^2^, the time range was 90 min. With the development of automated systems for body composition analysis that can be integrated into electronic medical record systems [42–45], skeletal muscle mass may be an actionable marker to predict patients at risk of prolonged operations and their associated complications. Because complications increase the time to adjuvant treatment, and longer time to adjuvant treatment is associated with shorter survival [46], consideration of skeletal muscle mass may help to improve overall outcomes for patients.

The specificity of the relationships between operative time and skeletal muscle suggests that operative time is not simply related to body size, with larger patients having longer surgeries, but instead, directly related to skeletal muscle mass. The musculature of the abdominal wall is often difficult to retract due to its stiffness despite the use of paralytics during surgery, and larger amounts of skeletal muscle may be more difficult to retract. Therefore, muscle mass may impact the time needed for intraoperative access to the peritoneal cavity and the amount of available operative space. Adipose tissue is more compliant and therefore more easily retracted, which may explain why only muscle mass was associated with operative time. Our data and the nature of this study are unable to directly explain why muscle mass is associated with operative time or why these relationships are inverse for males and females.

Like all studies, ours is not without limitations. Our retrospective study is limited to a single institution with a modest sample size. Given the nature of this study, there are likely additional factors we were unable to assess. Furthermore, our data can only demonstrate an association between muscle mass operative time and cannot determine that muscle mass is a driving factor in operation length.

## Conclusions

Our data demonstrate that measures of adiposity are not associated with pancreaticoduodenectomy operative time. However, consideration of sex and muscle mass may allow pre-operative identification of patients at risk of longer operations, leading to better outcomes for patients.

## Data Availability

The datasets used in the current study are not publicly available due to the relatively small sample size and risk of identification. They are available from the corresponding author on reasonable request and approval of the University of Iowa Institutional Review Board, as the original ethics approval did not include approval to publish individual datapoints.

## Abbreviations

BMI: Body mass index
BSA: Body surface area
OR: Operating room
CT: Computed tomography
PERCH: Patients Enhancing Research Collaboration at Holden
NCCN: National Comprehensive Cancer Center
ASA: American Society of Anesthesiologists
NSQIP: National Surgical Quality Improvement Program
ANOVA: Analysis of Variance
NIH/NCI: National Institutes of Health/National Cancer Institute
AJCC: American Joint Committee on Cancer
